# Countering Distrust in Illicit Online Networks: The Dispute Resolution Strategies of Cybercriminals

**DOI:** 10.1177/0894439321994623

**Published:** 2021-03-05

**Authors:** Benoît Dupont, Jonathan Lusthaus

**Affiliations:** 1 5622 Université de Montréal, Quebec, Canada; 2 6396 University of Oxford, United Kingdom

**Keywords:** cybercrime, dispute resolution, governance, closed forums and marketplaces, content analysis

## Abstract

The core of this article is a detailed investigation of the dispute resolution system contained within Darkode, an elite cybercriminal forum. Extracting the dedicated disputes section from within the marketplace, where users can report bad behavior and register complaints, we carry out content analysis on these threads. This involves both descriptive statistics across the data set and qualitative analysis of particular posts of interest, leading to a number of new insights. First, the overall level of disputes is quite high, even though members are vetted for entry in the first instance. Second, the lower ranked members of the marketplace are the most highly represented category for both the plaintiffs and defendants. Third, vendors are accused of malfeasance far more often than buyers, and their “crimes” are most commonly either not providing the product/service or providing a poor one. Fourth, the monetary size of the disputes is surprisingly small. Finally, only 23.1% of disputes reach a clear outcome.

In recent years, social scientific research on cybercrime has grown. One particular area of cybercrime scholarship that has blossomed is the study of the online organization of financially motivated cybercriminals. New types of data derived from cybercriminal forums and marketplaces may have spurred the growth of this niche field (see, for instance, Decary-Hétu & Dupont, 2013; Dupont et al., 2016; Holt, 2013; Holt & Lampke, 2010; Holt et al., 2015; Hutchings & Holt, 2015). Among these investigations, some scholars have begun to examine the issues of cybercriminal trust and cooperation (Holt, 2013; Motoyama et al., 2011; Yip et al., 2013). These studies generally examine the most structured elements of governance found in cybercriminal marketplaces, such as the feedback and rating systems that allow users to distribute information about others, the availability of escrow services, and the exclusion/banning of certain members. The puzzle of how cybercriminals navigate such a distrustful environment and successfully cooperate is an important one (see Lusthaus, 2018a, 2018b), as it helps determine their level of criminal achievement and by extension their ability to attract and inspire others to join in this cybercriminal activity (Tremblay & Morselli, 2000). But gaps remain in this literature.

There are two particular topics in this area that need to be explored in greater detail. First, many of the existing studies have been focused on more open forums, which are either public or allow anyone to register for an account, and data therefore are easier to access. Closed forums—which are vetted and therefore considered to hold a “higher class of cybercriminal”—have not been widely studied, as the data are much more difficult to access (for rare examples using closed data, see Dupont et al., 2017; Motoyama et al., 2011). Data showing how cooperation works in these high-level forums would be a major asset. Second, greater nuance on how these mechanisms work in practice needs to be provided empirically. Past studies of open forum data have successfully identified the existence of certain mechanisms that aid cooperation. But this is only part of the story. For instance, one might identify third-party guarantor systems on certain forums that should, in theory, enhance cooperation. But without further analysis, we cannot know how effective such systems are (Lusthaus, 2018b, p. 135). Just because the known tools could enhance cooperation does not mean that they will necessarily achieve that goal in practice. More reliable insights on the collaborative patterns observed on high-level cybercrime forums are particularly important to calibrate law enforcement interventions in these settings. Knowing how trust and distrust form within a cybercrime network and how they enable or hinder transactions could, for example, inspire interventions that would prevent the emergence of trusted relationships, would corrode existing ties—thereby increasing transaction costs—or would allow investigators to pass more credibly as cybercriminals by adopting similar behaviors (Décary-Hétu & Dupont, 2013; Franklin et al., 2007; Yip et al., 2013).

This article addresses these two gaps with a case study of Darkode, an elite cybercriminal forum that operated between 2007 and 2015. The U.S. Attorney of the Western District of Pennsylvania, David Hickton, who oversaw the investigation of Darkode described it in these terms:

Of the roughly 800 criminal internet forums worldwide, Darkode represented one of the gravest threats to the integrity of data on computers in the United States and around the world and was the most sophisticated English-speaking forum for criminal computer hackers in the world. (U.S. Department of Justice [USDOJ], 2015)

While some might quibble on details, Darkode was certainly a top tier forum in its day, particularly within the English-language scene. Part of this can be determined from its membership, which was exclusive and elite. While many open forums can have thousands of members, Darkode only had a few hundred. It also had a reputation for the trade of high-quality technical products and services, including exploit kits, botnets, malware, coding services, databases, and 0-day exploits (Lusthaus, 2018b, p. 50). By analyzing this Darkode data, we meet the first objective of engaging with higher level forums that allow us to study the behavior of more senior and sophisticated cybercriminals.

We meet the second objective by carrying out a detailed empirical analysis of these data. The core of this article is a rigorous investigation of the dispute resolution system contained within Darkode. Rather than describing the operation of multiple marketplaces at a macro level, we engage with a specific forum at the micro level. While the above literature tells us that a quasi-court process exists within a number of cybercrime forums, the nuances of how these arbitration systems function and their effectiveness are not known. Extracting the dedicated disputes section from within the marketplace, where users can report bad behavior and register complaints, we carry out content analysis on these threads. This involves both descriptive statistics across the data set and qualitative analysis of particular posts of interest.

This article proceeds in four sections. First, it outlines theoretical background on criminal cooperation, governance, and, particularly, dispute resolution. Second, it outlines the data and methods employed for the study. The third section examines the results of the analyses, while the final section offers a discussion of these results and ideas for future directions in cybercrime research.

## Theoretical Background: Criminal Cooperation, Governance, and Dispute Resolution

Before carrying out the case study of Darkode, it is valuable to engage with existing theory on cooperation, governance, and dispute resolution. In general, there appear to be a number of overlaps between the regulation of cybercrime markets and other more conventional criminal markets (Afroz et. al., 2013; Holt & Lampke, 2010; Leukfeldt et. al., 2020; Nurse & Bada, 2019). This makes it important to address theory that goes beyond cybercrime alone. For instance, when discussing those who govern criminal markets, this leads us to a discussion of organized crime groups and mafias, which is a key component of the existing literature (see Varese, 2010). There are also potential connections with the broader literature on the governance of legal marketplaces, which will be touched on in this background section.

Operating beyond the law, and without the protection of the state, creates a number of difficulties for successful cooperation, not least a need for secrecy and a lack of trust in others. Campana and Varese (2013) summarize the increased challenges facing criminals:

In the underworld, actors face more natural obstacles to be overcome. By definition, one cannot turn to the state to protect stolen or illegal assets. Information about the quality of goods and services is hard to come by, as there are no reputable and easily accessible sources of unbiased information. One cannot even be sure that the person offering a deal is not an undercover agent or a police informant. Regardless of personal inclination to cheat, actors in the underworld are difficult to locate, as they move around frequently. Entrepreneurs in these markets cannot freely advertise their good reputation, creditors disappear, informants consort with the police, and undercover agents try to pass themselves off as bona fide fellow criminals. (p. 265)

Humans seek order in their dealings, and criminals are no different. A number of scholars engage with the tension of how successful cooperation is achieved in extralegal contexts, where it often would be expected to fail (Campana & Varese, 2013; Gambetta, 1993, 2009; Levi, 2008; Reuter, 1983; Skarbek, 2011; Varese, 2001). In many of these cases, the governance of the underworld is a key concern (Schelling, 1971). When the state cannot or will not perform a governance role over certain activities, a new class of third-party protectors may emerge (see Dixit, 2004). One of the core tasks of these protectors is to keep order and ensure that their clients are not vulnerable to attack, theft, or extortion (Nozick, 1974, pp. 3–25). But, of equal importance, these men of violence help prevent disputes between their clients. Early in his study of the Sicilian Mafia, Diego Gambetta quotes a local cattle breeder who explains the importance of such third-party protection:

When the butcher comes to me to buy an animal, he knows that I want to cheat him. But I know he wants to cheat me. Thus we need, say, Peppe [that is, a third party] to make us agree. And we both pay Peppe a percentage of the deal. (Gambetta, 1993, p. 15)

Mafiosi perform this same “Peppe” role for criminals.

But sometimes disputes take hold and it remains the role of protectors to resolve them. This has been observed across a range of different criminal contexts and sometimes into the semilegitimate world too. Italian-American mobsters regularly arrange “sit downs” when members or clients come into conflict, which variously can lead to peace offerings or death (see episodes within Pileggi, 1986 and Maas, 1997). The Russian Mafia has run a shadow arbitration system used by criminals and noncriminals alike, who either can’t proceed through the official government system, or are deterred by the time required, the costs involved, or the threat of corruption (Varese, 2001). In Japan, the *Yakuza* has partly filled the void of the state, which failed to enact appropriate laws and provide enough legal professionals and other agents who can effectively enforce property rights (Milhaupt & West, 2000).

Dispute resolution has been observed in a range of other illicit settings, including in prisons, IRA-controlled neighborhoods, favelas, and the world of professional thieves (Arias & Rodrigues, 2006; Conwell & Sutherland, 1956; Hamill, 2011; Skarbek, 2011). One strong concentration of research has been around the drug trade. As this market has been criminalized, disputes must be dealt with informally, without the aid of the justice system (Black, 1983; Reuter, 2009). In the criminological literature, this variously has been termed “informal control” or “popular justice” and is generally seen to include four main elements: toleration, avoidance, negotiation (or informal mediation), and retaliation (Jacques & Wright, 2011). Within the street-level drug business, some research suggests the importance of retaliation for loss recovery and reputation management over the other avenues for approaching disputes (Topalli et al., 2002; see also Jacobs & Wright, 2006).

Much of the ability of drug gangs and broader organized crime groups to provide governance and resolve disputes is built on the threat of violence (Campana & Varese, 2018, p. 1393). This is what underpins the enforcement mechanism, even when negotiation or mediation is involved instead of direct retaliation. In practice, acts of violence might be relatively rare, as some groups can rely on a reputation for toughness to prevent them from actually having to carry out attacks (Campana & Varese, 2013; Gambetta, 1993).

This threat of violence, or the lack thereof, is potentially what distinguishes online cybercriminal dispute resolution from conventional criminal dispute resolution. Some cybercriminals operate off-line and, at least in theory, have conventional criminal dispute resolution avenues available to them (Leukfeldt et al., 2017a; Lusthaus, 2018b; Lusthaus & Varese, 2017). But in online settings, few enforcement avenues are available. One simple option is that cybercriminals refuse to continue working with those who have wronged them, leveraging an avoidance strategy used in many other licit and illicit settings (Black, 1993, p. 79; Dickinson, 2017, p. 8). But they can also raise the stakes by using tools that are more analogous to the use of violence, but largely in a weaker digital form. For example, Dupont (2014, pp. 32, 33) describes the use of distributed denial-of-service (DDoS) attacks, by those within a hacking group, as a punitive measure. Other enforcement tools are “doxing,” which involves publishing personal information on the targeted cybercriminals, and “swatting,” where a call is placed to emergency services and a tactical police unit is sent to the target’s location where it is suspected a violent crime is underway (Lusthaus, 2018b, pp. 130, 131). This latter tool manages to turn a virtual threat back into a kinetic one and therefore may be the closest equivalent to a violent attack itself.

Beyond the self-help and avoidance strategies outlined above, forums extend the enforcement options available to keep cybercriminals in line. As such, they are significant providers of cybercriminal governance. These online marketplaces suggest a possible fusion of self-governance and private governance (Dixit, 2004). In relation to self-governance, forums expand the reputation mechanism, so that information can be diffused much more widely. They broadcast this information in a more formalized way and allow for users to assess reputation at scale, rather than only in small networks. But the reality is not one of self-governance alone. Private governance also plays some role. Alongside the self-reporting mechanisms various marketplaces provide, forum officers ostensibly police against scamming, which brings them into compliance with aspects of the theory of protection (Gambetta, 1993, ch. 1; Nozick, 1974, pp. 3–25; Varese, 2001).

But while individuals in such forums perform mafia-like functions, it is unlikely the forums could classify as mafias themselves. This is largely because these forums are not criminal organizations at all, but are essentially marketplaces where otherwise autonomous groups and individuals come to trade (Lusthaus, 2013). In fact, while cybercrime forums are often presented as something new, it is likely that they follow the broad principles of how marketplaces of many kinds function. They are not dissimilar from legitimate online platforms like eBay (Dellarocas, 2003; Diekmann et al., 2009; Resnick & Zeckhauser, 2002), or even from off-line markets that have functioned throughout history (Greif, 1989; Milgrom et al., 1990). For example, the medieval Champagne fairs encountered comparable challenges to these modern day cybercrime marketplaces. These fairs were a major center of trade, where merchants gathered from many places across Europe. This presented some major issues of trust and enforcement, most famously examined by Milgrom et al. (1990):

At that time, without the benefit of state enforcement of contracts or an established body of commercial law, merchants evolved their own private code of laws (the *Law Merchant**)*** with disputes adjudicated by a judge who might be a local official or a private merchant. While hearings were held to resolve disputes under the code, the judges had only limited powers to enforce judgments against merchants from distant places. For example, if a dispute arose after the conclusion of the Champagne Fair about the quality of the goods delivered or if agreements made at the Fair for future delivery or for acceptance of future delivery were not honored, no physical sanction or seizure of goods could then be applied. (p. 1)

This appears to present similar obstacles to those faced by online markets. The solutions are not dissimilar either. Milgrom and coauthors argue that the Champagne fairs fostered trade because the private judges kept registers on the past behavior of merchants. When a merchant wished to conduct a new transaction, they would request information on their potential partner from a judge. This institutional reputation system allowed traders to avoid deals with those who had a poor track record. The private judges of the Champagne fairs could also exclude merchants from entry to future fairs, if they did not abide by their judgments. This provided an incentive to cooperate but also barred untrustworthy traders from participating.

In cybercrime, the policing role of forum officers to exclude “rippers” from the market acts as a deterrent to would-be scammers and also provides a broader assurance that the site is a safe location to carry out illicit commerce. This explains why the most widely reported sanction within forums is ostracism in the form of banning (Holt & Lampke, 2010; Morselli et al., 2017). But we lack a granular understanding of how cybercrime dispute resolution works. We have little detail on how the process functions, the full range of sanctions that are employed, and how effective these can be in a virtual environment. While cybercriminal trust/cooperation has attracted increasing levels of attention in the academic literature, distrust and failures of cooperation have been examined far less, despite their obvious policy implications for policing strategies that seek to disrupt illicit transactions, as an alternative or supplement to arrests and takedowns. For all the studies using forum data, detailed analysis of the “name and shame” sections and dispute resolution in these marketplaces has not been carried out. The rest of this article will address this void.

## Data and Methods

One of the major challenges faced by social scientists who research cybercrime is access to data that are both sufficiently comprehensive and accurately reflect the social dynamics of a shadowy community of individuals engaging in illicit activities. This is one of the main reasons why public forums dedicated to hacking and cybercrime topics have provided such a fertile and popular ground for data collection (see, among others, Dupont et al., 2016; Holt et al., 2015; Hutchings & Holt, 2015). They are the most easily observable layer of cybercriminal organization and are publicly accessible (see Lusthaus, 2019). They provide access to the online interactions of thousands of online offenders, who use them to learn new skills, trade in criminal products or services, or seek out potential co-offenders (Leukfeldt et al., 2017b). In other words, forums “provide [cybercriminals with] a highly visible point for networking” (Lusthaus, 2018b, p. 85) and offer criminologists a highly visible point for observing these networking patterns and trends.

While there is value in these data, there are also a number of methodological limitations associated with the study of public cybercrime forums. Social scientists often must develop their own technical expertise or find the funding necessary to hire the programmers who can build customized data collection tools (Pastrana et al., 2018). Many public cybercrime forums are populated by “lurkers”—members who never actively post or engage—and include a large number of participants whose criminal interests, expertise, and levels of engagement are fleeting (Benjamin et al., 2016). This results in patterns of exchange where the noise-to-signal ratio becomes problematic. In a study of the open-access Hackforums, which involved close to 450,000 pieces of “feedback exchange” over a 2-year period, Dupont et al. (2016) estimate that just 2.4% of the 30,000 forum members were responsible for 75% of the exchanges, in line with what has been observed on legitimate forums (Nonnecke & Preece, 2000).

The very accessibility and transparency of public forums may mean that their usefulness is limited in understanding how the upper echelons of the cybercrime community operate. To gather detailed data on this more elite group of cyber offenders who operate with greater secrecy, one must resort to time-consuming and hard to arrange interviews (Lusthaus, 2018b), to police files that may be difficult to access and may contain biases of their own (Leukfeldt et al., 2017b, p. 1398), or gain access to forums that are restricted to more advanced cybercriminals. The top forums usually require invitations from insiders and/or implement a vetting process that make it unlikely for an academic researcher to be able to secure access (Lusthaus, 2019). Ethical considerations also prevent social scientists from resorting to the deceptive practices used by law enforcement investigators, security analysts, and journalists to infiltrate these forums.

At present, the most effective way to gather data from higher tier forums is when the data have been made public. The fierce competition and internal feuds that fuel social interactions on these forums sometimes lead disgruntled members or hostile outsiders to hack them and leak their databases (Thomas et al., 2017). Leaked databases have been used in a small number of academic studies (Dupont et al., 2017; Motoyama et al., 2011). Many available database leaks are from publicly accessible forums and offer a more complete picture than can be scraped from online alone, for instance, possibly including private messages from within these sites. But there have been a small number of leaks from closed platforms too. In April 2013, a French hacker named Xylitol published a trove of files stolen from the English-language marketplace Darkode, which was at the time one of the most exclusive invitation-only cybercrime forums in the world (Dupont et al., 2017). These files can be downloaded from a public repository located at http://darkode.cybercrime-tracker.net. They are the main source of data for this article.

Although it is publicly available for anyone to download, particular care was taken in the ethical handling of this data set in order to minimize potential harms to forum users, from whom it was impossible to obtain informed consent. In line with the recommendations made by Holt (2010) and Thomas et al. (2017), safeguards were put in place, such as ensuring that no personally identifiable information was included (including logins and IP addresses) and that victim identifying information (such as samples of credit card numbers or bank account credentials) were also removed. The public Xylitol leak did not include private messages between forum members, which also limited opportunities for the disclosure of personal information.

The dismantling of the forum in 2015 by U.S. law enforcement organizations led to arrests and the shutdown of the site (USDOJ, 2015), so this is now a “closed” and somewhat historical case. This means there is a lesser concern of uncovering ongoing cybercriminal activity that might impact those individuals present within the data. When a specific file is cited, we use the file name that appears within the public database, each ending in “.png.” The user handles mentioned in this article were not anonymized. We do not believe this strategy threatens the users’ privacy, as there is no simple means to connect these handles with their true identity, except for those who were arrested and convicted and are now already in the public domain. It allows us to provide a better idea of how these participants wanted to be known to their peers (Gambetta, 2009). We believe that all these measures/factors protect forum users while furthering the public interest through allowing important research on how these elite cybercriminals operated (Martin & Christin, 2016).

The data used in this article are extracted from a set of 4,788 screenshot files that correspond to the forum’s discussion threads over a period of 4 years, from early 2009 to March 2013. Dupont et al. (2017) and Holt and Dupont (2019) have already examined the selection process implemented by the administrators of this exclusive forum. But no work has been done on the dispute resolution components of the forum, which are vital to understanding how, and how effectively, this forum was governed. To do this, we focus on a subset of forum threads that contain complaints and public accusations lodged by participants against some of their peers. This particular source of data allows us to measure the levels of distrust and conflict that remained within this community, despite its efforts to only admit the most reliable and capable members. Most importantly, it allows us to understand the system of arbitration employed to handle these disputes, how it operated, and how successful it was.

Members were directed by the Darkode administrators to lodge their complaints in a dedicated section of the forum appropriately called “scammers.” There were 263 files, which cover 160 disputes. This difference can be explained by the fact that a single discussion thread can extend over multiple pages, but each file (screenshot) in our database represents only one of those pages. We downloaded the 263 files that were found in the scammer section and coded them manually. It would have been more convenient to automate this process, but despite multiple attempts made in collaboration with computer science colleagues, it was not possible to use optical character recognition technology to facilitate the extraction and classification of the data. This was due to the format of the published data. The manual review and coding process allowed us to identify for each screenshot: (1) the nature of the complaint, (2) the nicknames of the parties involved, (3) the opinions expressed by various participants, including those not directly involved, and (4) the explicit or inferred outcome of the conflict resolution process. The coding process allowed robust content analysis, drawing on both qualitative methods and descriptive statistics.

This coding procedure had multiple phases. First, the codebook was built using an inductive process that extracted significant variables from an exploratory analysis of a random sample of 30 complaints and also relied heavily on schemes found in the hacker forum literature (Dupont et al., 2016; Holt, 2013; Lusthaus, 2018b; Morselli et al., 2017). Second, the codebook was given to two research assistants, each of whom was responsible for processing half of all the complaint threads. They then reviewed their counterpart’s coding to flag ambiguous statements or interpretations that could result in inconsistent coding, which were discussed and resolved during weekly meetings. When necessary, the codebook was updated and—if needed—past entries were revised in the database. Finally, the two coauthors independently sampled and verified components of the coding to ensure its accuracy and that there were no systematic errors.

This multiphased process ensured a good degree of robustness but also made clear that some subjectivity would always remain. This is a feature of many types of coding that have subjective categories (e.g., nature of complaints). But there were additional challenges of working with this particular data set, for instance, that the “hackerish” language used can be difficult to interpret or that fragments of key information may not be contained within the public posts. This meant that even certain ostensibly objective categories (e.g., financial losses) can take on a subjective component when coded. Furthermore, the forum administrators sometimes used deceptive practices—such as posting as guests—that could not always be detected.

## Results

Before we delve into the results in detail, it is important to briefly discuss the guidelines that were provided to Darkode users by the forum administrators. In an anonymous post titled “READ!!!” shared in July 2009, at the very beginning of the forum’s existence, and which remained at the top of the topics list, two of the administrators outlined the threshold of misbehavior above which complaints were warranted, explicitly encouraging members to first communicate with their counterparts in order to avoid frivolous accusations:

…I’ve seen more than one topic open without reasons, if you got complications while you are making deals, time delays, bad quality service/product or the shit that you bought wasn’t the shit that you were specting: Please, don’t be so childish and talk with the vendor the time that you need to talk, or in any case, don’t deal again with him.

I’ve already seen more than 6 posts calling “scammer” to people I know and I know they are legit since more than 3 years.

If someone took your money, and never finish his part of the deal, never replies, and is obviously fucking you. Feel free to post, only if you can prove it.

[…] Lack of communication is the main reason why people decide to accuse someone of scamming but this is not often the case, if you are sure you are scammed please make sure you include all details of the deal and provide evidence such as chat logs. If you would prefer to take the matter up in private, feel free to PM [private message] a Mod [moderator] or Admin. (READ!!!.png)

Members were strongly encouraged to provide as much evidence as possible to support their accusation and in particular excerpts of private conversations that could confirm an unfulfilled commitment or incriminate a business partner. These instructions confirm that the scammer section was not merely a subforum where members could vent their frustrations freely and disparage competitors but was designed by the forum leadership as a conflict identification and resolution platform that could help maintain the integrity of transactions conducted on Darkode and by extension sustain the forum’s broader reputation. Yet, it is important to note that not all complaints were dealt with in such a public manner. As was stated in the final sentence of the guidelines presented above, members also had the opportunity to reach out to a moderator or an administrator to launch a private complaint procedure. We do not have access to the private messages that were exchanged between Darkode members and have no way to assess what proportion of disputes were handled behind closed doors, compared to the public cases analyzed in this article. This is one of the limitations of our data, and future research should attempt to fill this knowledge gap.

As already indicated, the number of formal complaints made over a 4-year period reached 160. Although it is difficult to contextualize this number in the absence of comparative statistics from other cybercrime forums, this could be considered high when we recall that the main purpose of Darkode was to establish a trust network between an elite group of cybercriminals. It seems that, even in a top-level marketplace, the challenge of distrust among cybercriminals remained: The admission procedures that had been implemented to minimize the number of deceptive or incompetent members were not as successful as expected in stemming the flow of complaints. This may have led to the arbitration mechanism shouldering extra burdens in maintaining the successful functioning of the marketplace.

### Profiles of the Parties

The parties in the 160 disputes were 99 unique plaintiffs and 117 unique defendants, with 75 of these defendants being forum members. The Darkode database contained 348 profiles when it was breached by Xylitol in 2015. Using this number as an approximate baseline, 28.4% of members lodged a complaint and 21.6% were accused by their peers over our period of reference. This seems high for a community seeking to build trusting relationships. It would have been interesting to assess whether complaints were concentrated on the most active profiles, but the format of our data did not allow this type of analysis, as it is challenging to infer levels of activity from these screenshots.

Of the 99 plaintiffs, 70 (70.7%) made a single complaint while 29 (29.3%) made multiple complaints. Multiple complainers were responsible for 54% of accusations, indicating a disproportionate role in litigating unwanted behaviors on this forum. The two most prolific complainers made 11 formal accusations each, while the four administrators rank among the 10 most active accusers. Administrators alone, who constitute only 4% of complainants, are responsible for 13.2% of complaints, most of them as a result of personal transactions gone wrong and perhaps reflecting that they themselves transacted very regularly on the forum.

Among the 117 defendants, 91 (77.8%) received a single complaint while 26 (22.2%) faced multiple accusations. The most implicated member received eight complaints (six under his main alias and two under a secondary identity he used less regularly), while his “runner-up” was accused 6 times under five (linked) identities. The 26 members who received more than one complaint collectively accounted for 42.5% of complaints, reproducing a similar concentration found among complainants. The two main differences are: (1) the accumulation of identities, which in the end did not seem to shield returning wrongdoers from the ire of their peers, who often figured out a scammer had returned under a new nickname; and (2) the underrepresentation of higher ranking members in the ranks of defendants.

Table 1 shows the distribution of plaintiffs and defendants by hierarchical level in the formal system of ranks and privileges designed by the creators of Darkode. At the bottom of the pyramid were *Guests*, who were granted temporary access to the marketplace but not other sections of the community, and could introduce themselves and make a case for their permanent admission. Those who were accepted—a vast majority of candidates—then acquired the status of *Fresh Fish* and would progress to *Level 1* and *Level 2* as they acquired a track record and became more trusted by their peers. The most committed could play the role of *Moderator*, helping the *Administrators* monitor and regulate discussions on Darkode’s various subforums (Dupont et al., 2017). As noted, a number of complaints were also made against individuals who were not members of the forum (but who operated on other underground markets) or whose status was impossible to establish with certainty. The fact that warnings were issued against traders from other forums illustrates how reputation is not completely tied to a particular site but is viewed as a currency that is traded on the broader underground, at least for those diversified high-profile users (Frank et al., 2018). Meanwhile, in the “unknown” category were, for example, individuals who seemed to have traded on Darkode but for whom we could not find any matching record in the members’ database.

**Table 1.** Distribution of All Complaints Made According to the Status of the Plaintiff and the Accused.

Status of the Accused								
Status of Plaintiff	Unknown (%)	Nonmembers (%)	Guest (%)	Fresh Fish (%)	Level 1 (%)	Level 2 (%)	Administrator (%)	Total (%)
Suspended	0	0.6	1.2	0	0	0	0	1.8
Guest	20.0	1.9	25.0	1.3	1.2	0	0.6	50.0
Fresh fish	3.7	0	6.3	0.6	1.3	1.2	0	13.1
Level 1	2.5	1.2	10.0	1.3	0.6	0.6	0	16.2
Level 2	1.9	0.6	1.3	0	1.3	0	0	5.1
Moderator	0	0	0.6	0	0	0	0	0.6
Administrator	3.8	1.3	7.5	0.6	0	0	0	13.2
Total	31.9	5.6	51.9	3.8	4.4	1.8	0.6	100.0

There was a clear concentration of complaints against the lower echelons of this community with guests and cybercriminals with an unknown status receiving more than 83% of accusations, while administrators and Level 2 members were only incriminated in less than 3% of disputes. Even Fresh Fish and Level 1 members seemed relatively sparred, having received one third to one fourth less accusations than the number of complaints they lodged against others—essentially guests and participants with unknown status. It would be incorrect to define this dispute resolution system as unidirectional, since guests also account for half of all complainants and are therefore able to seek redress when they feel they have been abused, but it remains obviously asymmetrical. This seems to confirm the regulatory function played by this dispute resolution mechanism, which is mainly directed at the entry-level participants of this forum and enables their further screening for unreliability and untrustworthiness.

### Nature of the Complaints

The types of complaints made can be divided into the 10 categories listed in Table 2. They reflect business or technical failures, as well as general warnings against a particular vendor or buyer. The first observation that can be made is that sellers were accused more often than buyers of not respecting the terms of the agreement between the two parties. At least 57.6% of complaints concern a seller, while only 7.5% of accusations were directed at a buyer. The remaining 32.8% complaints were of a more general nature about the unreliability of a forum member with limited details on their trading status.

**Table 2.** Distribution of All Complaints According to the Nature of Disputes.

Type of Dispute	Frequency	%
Seller took the money and didn**’**t deliver the product	35	21.9
Warning about a member’s unreliability (because of the use of multiple identities, a perceived lack of knowledge, untrustworthiness, excessive pricing policies, etc.)	24	15.0
Seller sold defective or incomplete products	23	14.4
Unspecified	17	10.6
One of the two parties did not abide by the terms of the deal	16	9.9
Buyer took delivery of a product but didn’t pay (or failed to pay the full amount)	12	7.5
Seller distributing a product without the authorization of the author or reselling free products	12	7.5
Seller took money and refused to refund	10	6.3
Seller did not provide adequate or expected customer support (including delay of responses or disappearance)	11	6.9
Total	160	100

The most frequent accusation (21.9% of disputes) is directed at sellers who fail to deliver a product or a service such as customized malicious code or bulletproof hosting services, despite having received payment. The transfer of money is usually made to the seller following a detailed conversation about the technical specifications of the product to be delivered, which serves to reassure the buyer, but communications slow down or cease entirely after the payment is received. The seller then becomes unresponsive or disappears from Darkode.

The delivery of defective or incomplete products or services represents the second most frequent type of seller-focused dispute (14.4% of disputes). A typical accusation was the one launched by *d0laR* against *nocen*, a former Darkode administrator: “I paid him 3k+ for a project that he didn’t finish. And he blocked me since then (1+ year ago)” (nocen.png). Given that some kind of product/service was provided in these instances, the main difference with the previous configuration is that a higher level of uncertainty sometimes remained regarding the good faith of the accused. This led participants into extended conversations about their past positive or negative experiences with the accused and questioning of the role played by the complainant in the deal’s collapse, highlighting, for example, a possible lack of skills to handle the product, a constant change of requirements, or unrealistic expectations from the buyer. When *Dario* complained about the high failure rate of a botnet of 10,000 infected machines he had purchased from an associate of *Abra Kadabra*, participants were quick to point out that considering the low price paid to the provider, quality was never going to be very high and that “u must not have much experience with bots to expect” high levels of stability (Installs Abra’s Friend Referal x1.png). Failure to complete a functioning product was also sometimes caused by circumstances beyond the seller’s control, such as an arrest. In May 2012, a complaint was lodged by *nominator* against *Arkham*:

Took couple of hundreds from me and provided some broken stuff. He seems dedicated at the start but after this he always disappears making excuses. Please ban him if he doesn’t provide any explanation. Thank you. (Arkham x1.png)

When two members (*dexter* and *Tux*) mentioned *Arkham* had been arrested, and that a third one (*LeNo*) even suggested he could be dead, the discussion came to an end, although the plaintiff still requested that the defendant’s account be disabled “for security measures.”

Meanwhile, 7.5% of accusations involved sellers distributing products without authorization from their designers or products that were freely available on other forums. For example, *JohnHoudaille* complained against *trukovn* on the following grounds:

I sold him a blackhatworld vip account (for $20), told him about some cool scripts in that section, so what does he do? 10 hours later hes selling the code on this forum here. (trukovn == Lammer x1.png)

The concern here is that such practices undermine the forum’s reputation by devaluing the quality of its products and by dissuading elite malware creators from trading on it, for fear that their products will be leaked and that they won’t be able to profit from them.

Finally, the quality of customer service provided by sellers was not always as satisfactory as purchasers expected. Refunds were not always provided when requested (6.3% of accusations), and limited or no assistance was available when products or services stopped working or did not perform as advertised (6.9% of accusations). For example, *clientsm* expressed his frustration at the marketers of the botnet Hades:

Hello, I am writing here this detailed information about hades bot and its functions that this bot is not what they say in the sales thread. […] the reality of this bot is it’s a non working beta version and after you have paid the owner will send people to help you but he himself wont send you msg [messages]. After this drME [the accused] stopped talking to me and I keep msging [messaging] him whole day he says I need to go, I need to sleep, I need to catch this, I need to go there. I don’t know how he can do business when someone paid him $$ and waiting for him all day to come and he wont come back and help. (Hades Bot x1.png)

The complaints against buyers failing to pay for the products or the services they procured on Darkode (7.5%) were usually straightforward and circumstances are relatively similar to the reverse situation where sellers fail to deliver the product or services for which they have received payment. In this case, a seller is approached by a buyer who enquires about a product or a service, explains their needs, and promises to pay on delivery. Back and forth discussions are generally conducted to assess the needs of the client and his satisfaction once the first part of the deal has been completed. Buyers then start to become unresponsive when sellers ask for their payment or block them entirely from their communication channels.

Beyond specific disputes about aborted or incomplete transactions, the second largest group of complaints (15% of accusations) did not seek to settle a particular incident but instead tried to provide a general warning about a particular member’s perceived untrustworthiness to the rest of the Darkode community. *Gonzo* shared his concerns about the sudden and unannounced disappearance of *exchange*:

Everyone be careful. He [*exchange*] hasn’t been online in 2 days. And people on other forums are telling me that they sent him mtcn;s [Money Transfer Control Numbers, a tracking number issued by Western Union with any money transfer]. He picked up and has not replied yet. (exchange—POSSIBLY.png)

Members were also singled out for sharing Darkode posts on other forums (sometimes to inflate their reputation), for sharing their account with other members or guests, for leaking malicious software or databases sold exclusively on Darkode, for assuming multiples identities that sometimes pursued opposing interests, and in general for deceiving other members. We also find in this category warnings about scammers who do not belong to Darkode but are very active on other forums. In these cases, the complainant provided identifying information such as a nickname, email addresses, instant messaging account handles, chat logs, and so on, in order to dissuade others from doing business with the accused.

In most categories of conflicts, multiple interpretations of what constituted a reasonable response time were discussed and unexpected circumstances such as a police arrest or a medical emergency were offered by third parties to the conflict as an alternative explanation for the disappearance of a forum member. Very often, two rationales collided in these conflicts: For some, delays and hiccups were experienced as the unavoidable costs of doing business with unreliable strangers in an unstable criminal environment. This pessimistic outlook often served to justify why preliminary precautions needed to be taken to assess a partner’s trustworthiness prior to any deal. Others brought a more business-oriented mindset to the forum and expected transactions to proceed swiftly, analogous to what could be expected on more conventional marketplaces. An anonymous guest summarizes this second approach in a dispute between *Dario* and *pi0neer*, where it is suggested the offender might have been “busy.”

That’s bullshit, I don’t care how busy someone is if they initiate the transaction by accepting the product or service it becomes priority in my opinion until the deal is fulfilled on their end. (pi0neer x1.png)

The categories outlined above are porous, while the same person could be a source of complaint both as a seller and a buyer. The scammer label could also be attached to someone who had previously completed a significant amount of trades with high levels of reliability. An illustration of this is the complaint lodged against *Palgue* by *Fcorp* for failing to refund him. In the ensuing discussion, a third party, *sumadinac* chimed in, stating that *Palgue* owed him money for the purchase of 30,000 compromised computers. But when an administrator added *Palgue* to the list of members with a bad reputation, he was quick to defend himself and to remind the plaintiffs that he had conducted many prior transactions without any complaint and that exceptional circumstances (probably a downturn in business) were causing these payment or refund delays. His argument seemed to convince *Fcorp* who closed the thread on the following injunction: “Palgue, move yur ass, I need my money quickly!” (Palgue.png)

Contingency tables were used to analyze the frequency of various types of complaints made by plaintiffs based on their status in the forum (Table 3), as well as the frequency of various types of complaints based on the status of the accused (Table 4). These two tables reinforce the observation made in the previous section regarding the concentration of complaints among entry-level participants: Guests are uniformly overrepresented across all complaint categories, both as complainant and accused, whereas Level 1, Level 2, and administrators only received a couple of complaints in a limited number of categories. The only exception to this skewed distribution toward the lower end of the forum’s hierarchy is the significant number of public complaints lodged by administrators across all categories, despite their ability to suspend or ban members without any consultation. As was shown in Table 1, all of these complaints were directed at guests or accused whose status was unknown.

**Table 3.** Type of Complaint by Status of Plaintiffs.

Nature of the complaint	Suspended Member	Guest	Fresh Fish	Level 1	Level 2	Moderator	Admin	Total
Took the money and did not deliver product		12	5	12	1	1	4	35
Warning about a member unreliability	1	9	3	1	1		9	24
Seller sold defective or incomplete products		12	4	5	2			23
Unspecified		13	1				3	17
One of the two parties did not abide by the terms of the deal		10	4		1		1	16
Buyer took delivery of a product but didn’t pay (or failed to pay the full amount)		5	1	4	1		1	12
Seller distributing a product without the authorization of the author or reselling free products	2	5	1	1	1		2	12
Seller took money and refused to refund		6	2	1	1			10
Seller did not provide adequate or expected customer support (including delay of responses or disappearance)		8		2			1	11
Total	3	80	21	26	8	1	21	160

**Table 4.** Type of Complaint by Status of the Accused.

Nature of the complaint	Not a Member	Guest	Fresh Fish	Level 1	Level 2	Admin	Unknown	Total
Took the money and did not deliver product	2	20	3	1			9	35
Warning about a member unreliability	2	11	1				10	24
Seller sold defective or incomplete products	2	10		3			8	23
Unspecified		10					7	17
One of the two parties did not abide by the terms of the deal		5	1	2	2		6	16
Buyer took delivery of a product but didn’t pay (or failed to pay the full amount)		9					3	12
Seller distributing a product without the authorization of the author or reselling free products	2	5		1	1		3	12
Seller took money and refused to refund		7	1				2	10
Seller did not provide adequate or expected customer support (including delay of responses or disappearance)	1	6				1	3	11
Total	9	83	6	7	3	1	51	160

*Note.* Moderators did not receive any complaint.

### Financial Losses

Estimating the overall costs of cybercrime is an area fraught with difficulty and divergent agendas (see Anderson et al., 2013; McGuire, 2018). At a granular level, there are journalistic accounts suggesting that cybercriminals lead lavish lifestyles (Goodin, 2019; Wilber & Strohm, 2015), but there is a paucity of scholarly data regarding the specific profits generated by particular cybercrime operations. Holt et al. (2016) have attempted to estimate the revenues generated by stolen data sellers on illicit online markets. They highlight the difficulty in measuring the volume of transactions and their final amount, in contrast to the prices advertised. This is because the vast majority of sales is finalized over private messaging systems. Nonetheless, they settle on high estimates ranging from tens of thousands to millions.

By surveying the financial losses claimed by the plaintiffs in the scammer section, our analysis offers another way of partially addressing this subject. We were able to extract the amounts involved in 74 of the 160 disputes analyzed and found that the mean loss claimed by a complainant was US$1,175, while the median loss was a paltry US$300 and the mode an even smaller US$100. The range extended from US$20 for access to a VIP account on a competing hacker forum to US$18,000 for a share of a hack into a cryptocurrency account. Although there is no reliable formula that would allow us to estimate the average size of transactions based on the average losses claimed by complainants, or to estimate the overall earnings of individual users, these amounts seem low for a supposedly elite marketplace.

### Outcomes of the Conflict Resolution Process

The adjudication process followed a relatively ad hoc and unstructured format. Once a complaint had been lodged publicly, the forum membership engaged as a community with the case, based on the information provided to them. No independent arbitrators were formally appointed by the forum administrators to guarantee the integrity of procedures. Evidence—usually screen captures of supposedly incriminating chat exchanges between the parties—could be posted on the discussion thread by anyone and would then be commented upon by anyone. The plaintiff usually drove this component of the process. Defendants were not excluded from the proceedings and chose to provide their side of the argument in 38.1% of disputes. Darkode members who knew or had conducted business with either the plaintiff or the accused also frequently intervened, even if they had played no role in the particular transaction that led to the complaint. As we have already noted, discussions were often animated and each complaint generated on average 16.8 posts from other members (range of 0 and 67).

Administrators got involved in 76.9% of complaints with a dual role: In 47.9% of disputes in which they intervened, they positioned themselves as adjudicators or mediators, while in the remaining 45.5%, they adopted a more hands-off approach where they merely stated their opinion without driving the resolution process and therefore no clear arbitrator took control. In a minority of cases (6.5% of the complaints in which they took part), administrators disagreed on their interpretation of the dispute. So, if we add the number of complaints they ignored with those in which they deliberately limited their role, 68.6% of disputes had to be resolved with no or limited assistance from the forum administrators. This is significant because administrators have exclusive control over enforcement mechanisms such as the ability to ban a member from the forum or to suspend membership temporarily. But one might also surmise that these disputes were considered less deserving of administrator attention, as they often concerned guests or nonmembers, or were of a general nature (e.g., community warnings).

There was a range of outcomes within the spectrum of disputes: The accused were banned from Darkode in 12.5% of disputes. In 9.4% of cases, a reparation (usually a reimbursement) was imposed on the accused and enforced by a forum member or an administrator, while a suspension was ordered in 1.2% of disputes, though given no time period was stated it is unclear if this category differed greatly from permanent bans. But it was impossible for us to determine a clear outcome for 76.9% of complaints. This likely included some disputes where the plaintiff was “innocent”, but where an “acquittal" outcome was not definitively stated. Table 5 presents the frequency of outcomes based on the types of complaints, showing that reparations are mainly used when sellers are accused of not having delivered what was expected or fail to reimburse unsatisfied customers. By contrast, bans are used more broadly across all complaint categories.

**Table 5.** Outcomes by Type of Complaint.

Nature of the complaint	Suspension	Reparation	Ban	Unknown	Total
Took the money and did not deliver product	1	7	2	25	35
Warning about a member unreliability			5	19	24
Seller sold defective or incomplete products		3	3	17	23
Unspecified			3	14	17
One of the two parties did not abide by the terms of the deal			1	14	15
Buyer took delivery of a product but didn’t pay (or failed to pay the full amount)	1		3	8	12
Seller distributing a product without the authorization of the author or reselling free products			1	11	12
Seller took money and refused to refund		4	1	6	11
Seller did not provide adequate or expected customer support (including delay of responses or disappearance)		1	2	8	11
Total	2	15	21	122	160

Complaint outcomes were classified as unknown either because no formal decision was published or because discussions veered off course and no resolution eventuated. In the second case, inflammatory posts often created opportunities for new accusations to be made that had little to do with the initial complaint. Matters quickly deteriorated into protracted exchanges of insults and sarcasm. Administrators either chose to ignore these discussion threads, hoping that participants would rapidly lose interest, or sometimes preferred to close them in an attempt to maintain the smooth functioning of the scammer section, and perhaps the decorum of the site as a whole. Such a preventative approach was adopted and formulated very clearly by one of the administrators in a conflict between *Segadora* and *d0lar*, the latter being accused by the former of having leaked his code. Five hours after the initial complaint had been lodged and confronted with mounting evidence of a broader conflict erupting, a final post appeared at the bottom of the thread: “Locked to prevent flame war.^1^ Everyone involved from both sides, grow up” (d0lar.png). On other occasions, threads were not derailed by insults but by a misplaced display of humor that interfered with the dispute assessment and resolution process. In a complaint about a seller who disappeared without having completed his side of the deal, exchanges flared up following what appeared at first as an innocuous comment on the irony of the situation:

*fubar*: that guy is also known as madz or m0deration or unknown3d […] ive heard that hes tried to screw me over before so I don’t deal with him and I recommend that nobody else does either :)

*birdy9*: “cr1mepayz” oh the ironyyyyyyyyyyyyyyyyyyy

*eth0*: you fucking dumb fuck stop making such comments in every thread. If you don’t have anything smart to tell then shut ur mouth. First you seemed nice person to me with a sense of humor but now I see why u get banned on every forum

*birdy9*: who are you? […] Guess you can’t handle when someone jokes around, I thought it was kind of humorous that someone with that msn name was also scamming criminals.

None of the following messages in the thread stayed on the topic of the initial complaint, with people instead questioning *birdy9*’s credentials. Despite this high level of uncertainty about the outcome of three quarters of the conflicts, which seem to linger or to remain unresolved, we should not discount the existence of informal sanctions being experienced by members named in formal complaints. We do not have the data to assess the extent to which the mere fact of being called out by a complainant might hurt one’s reputation and result in the loss of business opportunities, but this is a real possibility that should be investigated in future papers. It is also possible that some forms of retaliation and negotiation took place through more private communication channels, outside of this scammer thread.

## Discussion and Conclusion

The outcomes of this study largely confirmed theoretical expectations. The administrators and moderators of Darkode regulated the trade on the site with a degree of success, and dispute resolution was an important component of this. Whereas research into the off-linea drug trade suggests that retaliation is a key component of how disputes are addressed, in a virtual setting without the avenue for physical violence, this is much more challenging. Darkode showed that dispute resolution could take on a larger role in such a setting. Perhaps aided by a digital record of behavior, a semiformal system of mediation came to prominence. But with that said, and as discussed below, the system was far from perfect, and many users were still left simply to avoid future dealings with malefactors or to tolerate the reality of being “ripped” from time to time. This, too, is not out of line with what theory would suggest.

The results outlined in the previous section reveal some new details about dispute resolution in an elite cybercriminal forum. First, the overall level of disputes was quite high, even though members were vetted for entry in the first instance. Second, the lower ranked members of the marketplace were the most highly represented category for both the plaintiffs and defendants. Third, vendors were accused of malfeasance far more often than buyers, and their “crimes” were most commonly either not providing the product/service or providing a poor one. Fourth, the monetary size of the disputes was surprisingly small. Finally, only 23.1% of disputes reached a clear outcome.

These results are a mixture of what might be expected and what might be surprising. For instance, the fact that many disputes occurred among lower ranked members is intuitive in a number of ways. Lower ranked members were often newer to the forum and had also accrued less reputation within the community. They were likely to be less trustworthy both because they were yet to prove themselves and move up the ranks, and precisely because they had not built up such a brand, they had less to lose if they engaged in “ripping.” On the victim/plaintiff side, it is also possible that the relative inexperience of these lower rung members meant they were less attuned to selecting suitable deals and trustworthy partners. In reverse, it is also intuitive that higher ranked members less commonly appeared within disputes, both because they had more to lose by ripping and had proved their trustworthiness over time. But this might also be because their high standing in the community may have deterred more junior members from making accusations against them. That is, any malfeasance they carried out was less likely to be called out for fear that there might be consequences for the junior members. This view finds possible support in that most accusations against higher ranked users came from those who were also of relatively high rank.

It is also in keeping with expectations that most complaints would be against sellers rather than buyers. It is likely that, in order to protect themselves from a higher rate of scams, many sellers required payment in advance of delivery—a topic of occasional discussion in the dispute threads. This then put vendors in an opportunistic position where they were better placed to carry out scams themselves. The other factor likely driving a lower rate of complaints against buyers was that their role was generally to provide payment. In many cases, this was a binary: Payment was made or it was not. While one might think of instances of partial payment, there was generally going to be less disagreement whether the terms have been met than if a product or service was considered imperfect in some way, which would be far more subjective.

On the other hand, some findings are quite surprising. For one, the monetary amounts in the disputes appeared unexpectedly trivial for a marketplace with an elite reputation, where 0-day exploits and innovative new malware were being traded and where successful members were reportedly earning millions of dollars. One of the highest achievers in this community was Paunch, a 20-something Russian cybercriminal who reported to have generated US$50,000 in monthly revenues by selling exploit kits and crypting services on Darkode and other forums. Paunch joined Darkode in January 2011 and was arrested by the Russian police in October 2013 (Goodin, 2013). It may also be useful to remind the reader here that Darkode was rated by the law enforcement officials who supervised its takedown as the most dangerous cybercrime forum in the world, where the “most prolific cyber criminals” converged (Federal Bureau of Investigation, 2015). Yet the monetary figures found in Darkode’s Scammers section would seem more appropriate for a beginner forum, where aspiring cybercriminals are honing their skills and building a customer base.

There are a number of potential explanations for this puzzle. It may be that despite their successes, members remained relatively petty. Or to put in more charitable terms, they thought it was important to report misbehavior and seek justice even when the specific harm was limited. Such actions may have improved the functioning of the marketplace as a whole, and it was not particularly costly to instigate a grievance by a simple post. Linked with this is that cybercrime is largely a volume business. So while, for instance, an individual vendor may have been making significant sums of money, this may have been achieved through a large number of transactions. This means there was a much greater likelihood of a rip taking place in a smaller transaction. A related idea is that, even on an elite forum, only a relatively small number of users could be in the top echelon. Not everyone was likely to be making millions of dollars. One could argue that the more successful members of Darkode managed their business more astutely and were rarely exposed to these risks, which is why they were not being represented as widely in the Scammers section. Our data show that most of the disputes involved low-level players, which likely explains why the linked monetary figures were also lower. It is also possible that these (often newer) lower ranked users were testing the dispute resolution system of the marketplace and/or learning about the trust dynamics on the site (on reputation management in illicit forums, see Motoyama et al., 2011; Odabaş et al., 2017).

But taking an opposing position, one could surmise that a forum like Darkode was simply not as elite as people think. This argument links to the other main finding that appears counterintuitive: That on such a supposedly elite forum, the clear majority of the disputes were not resolved. Thus, not only were the member selection and vetting processes implemented by the forum administrators unreliable—as they still allowed a significant number of scammers inside—but the mechanism to handle the resulting disputes was also suboptimal. It seems the problem of distrust was not limited to lower tier forums. One could argue that the Darkode administrators never managed to create the type of trust network that characterize successful organized crime enterprises (Tilly, 2005).

There may be some truth to this argument. Cybercrime creates extra challenges for cooperation, beyond the instability of dealing with criminals alone. In online settings, anonymity is ostensibly guaranteed and physical enforcement becomes extremely challenging (Lusthaus, 2012, 2018a). It should not be surprising that substantial levels of distrust remain even after mechanisms are employed to reduce them to a lower amount (Lusthaus, 2018b, pp. 138–140). Within Darkode, various members appeared to acknowledge this reality. One argument was that the aspects of the business around monetization, which are generally considered blacker and less sophisticated, were threatening a forum historically known for more technical elements like malware:

*mafi*: when you start handing out bank accounts to random people you should be prepared to get scammed 90% of the time just fyi

*styler*: That’s why this forum should only stay as malware marketplace, not carder board :-)

*SnakeDeye*: so true in banking cashout business 90% of ppl are scammers. (hkg@hotmail.com x1.png)

For some, the reality of working in cybercrime was obvious. As *dice* put it:

This again proves that doesn’t matter how tight is this forum on who invite who, everyone can scam everyone. That is why this game is so great :-). (Gaza Nova x1.png)

This is in keeping with the view that cybercrime marketplaces are only one element of a broader industry and, for all their networking benefits, they are an element that involves some compromises. These forums, and their systems of governance, effectively scale up trust from small groups to hundreds or thousands of members, so that strangers can do criminal business together. Given this still involves large numbers of faceless users engaging in illicit transactions, significant distrust is likely to remain. This is why some (even more elite) cybercriminals choose to stay away from marketplaces and gather in much smaller, closed groupings (Lusthaus, 2019). It is likely also why offline interactions/organization appeal to some offenders (see Leukfeldt et al., 2017a; Lusthaus, 2018a; Lusthaus & Varese, 2017).

But while Darkode provided a system of governance that was suboptimal, perhaps it should not be judged too harshly. As with the Champagne Fairs and other examples throughout history, these individuals developed mechanisms that to a substantial degree did regulate their dealings in a very challenging environment.^2^ Part of their success may not have been only around banning untrustworthy members from future dealings but also providing a record of past misbehavior so other traders could take this information into account when choosing future partners and how to interact with them. As this was a “scammer” subforum, rather than a pure dispute resolution subforum, it was possible that part of its function was viewed as simply to transmit information on rogue actors to the community rather than producing a litigated outcome in all cases. This may be linked to the apparent lack of formal “acquittals”, which skews the rate of clear outcomes downwards. Instead, the emphasis was on identifying untrustworthy actors and sanctioning them in the more extreme cases.

These factors may help explain why successful outcomes on Darkode sat at less than 25%. But it’s not entirely appropriate to rate the forum’s effectiveness at regulation in absolute terms. Some comparison is required to understand what the level of dispute resolution actually means. This is very difficult as data are sparse. Dispute resolution has not been a widely investigated topic within cybercrime forums to date. While one could attempt to compare a grayer and less elite site, like Hackforums, these more open forums often don’t have a scammer section at all. Merely by having a scammer section, this qualitatively suggests that marketplaces like Darkode are governing themselves more seriously. But it is challenging to make a quantitative comparison in such cases. Another alternative is to compare cybercrime to conventional organized crime. While cases of successful governance have been analyzed, this is often done qualitatively rather than quantitatively (for instance, see Gambetta, 1993; Varese, 2001). This again makes a cross-comparison difficult and is a rare example of where cybercrime data are perhaps in more plentiful supply than another subfield. There remains large scope for future research to engage further with the subject of cybercriminal governance. But a core part of this effort must concern how best to overcome the data and methodological challenges of measuring the effectiveness of these governance mechanisms.

## Notes

1. An extended exchange of offensive messages.

2. One should also not overlook that, even within legitimate society, rates of successful dispute resolution are not commonly high. In both criminal and civil settings, only a fraction of cases ever make it entirely through the legal system, with very many cases remaining unreported, unresolved, or more informally concluded (see, for instance, Eisenberg & Lanvers, 2009; Home Office, 2018; Smith & Macqueen, 2017).
